# Influence of an energy deficient and low carbohydrate acute dietary manipulation on iron regulation in young females

**DOI:** 10.14814/phy2.15351

**Published:** 2022-07-03

**Authors:** Nanako Hayashi, Aya Ishibashi, Ayame Iwata, Haruka Yatsutani, Claire Badenhorst, Kazushige Goto

**Affiliations:** ^1^ Graduate School of Sport and Health Science Ritsumeikan University Kusatsu Shiga Japan; ^2^ Department of Life Science The University of Tokyo Meguro Tokyo Japan; ^3^ School of Sports, Exercise and Nutrition Massey University Auckland New Zealand

**Keywords:** hepcidin, iron status, low carbohydrate availability, low energy availability

## Abstract

Hepcidin is a liver‐derived hormone that regulates iron metabolism. Recent studies suggest that an energy‐deficient diet or low carbohydrate (CHO) availability may increase hepcidin in the absence of inflammation. The purpose of the present study was to examine the impact of either an energy‐deficient diet or an ED diet with low CHO intake during three consecutive days on hepcidin responses, hematological variables, and energy metabolism in young Japanese women. Twenty‐two young females were divided into two different groups, either an energy‐deficient with low CHO intake group (ED + LCHO; 2.0 ± 0.3 g/kg/day CHO, 39%CHO, 1123 kcal/day) or an energy deficient with moderate CHO intake group (ED; 3.4 ± 0.3 g/kg/day CHO, 63%CHO, 1162 kcal/day). During the three consecutive days of the dietary intervention program, participants consumed only the prescribed diet and maintained their habitual physical activity levels. Body composition, substrate oxidation, iron metabolism, and inflammation were evaluated pre‐ and post‐intervention. Serum iron and ferritin levels were significantly elevated following the intervention (*p* < 0.001, *p* = 0.003, respectively). Plasma interleukin‐6 (IL‐6) levels did not change following the intervention. Serum hepcidin levels significantly increased after the intervention (*p* = 0.002). Relative change in hepcidin levels was significantly higher in the ED + LCHO (264.3 ± 87.2%) than in the ED group (68.9 ± 22.1%, *p* = 0.048). Three consecutive days of an energy‐deficient diet increased fasting hepcidin levels. Moreover, elevated hepcidin levels were further augmented when an energy‐deficient diet was combined with a lower CHO intake.

## Graphical abstract

Three consecutive days of an energy deficit dietary intake increased fasting hepcidin levels. Moreover, elevated hepcidin levels were further augmented when an energy deficit dietary intake was combined with a lower CHO intake.

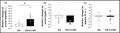

## 1 INTRODUCTION

Anemia is a common nutritional diagnosis, affecting ~29.9% of women of reproductive age (aged 15–49 years) globally (WHO, 2019). Approximately 19% of Japanese women of reproductive age are estimated to suffer from anemia (WHO, 2019). Although multiple factors contribute to the cause of anemia, iron deficiency anemia (IDA) is a major one (Cappellini et al., 2020). Iron is an indispensable element in the human body, due to its association with oxygen delivery, DNA synthesis, and cell respiration (Beard, 2001). As such, iron deficiency has been associated with impaired endurance exercise performance and developing the symptoms of fatigue, lethargy, and negative mood states (Sawada et al., 2014; Sim et al., 2019). A negative iron balance is one of the causes of iron deficiency, where daily iron losses and utilization may exceed daily iron intake (Cappellini et al., 2020). Additionally, impaired iron metabolism, where iron recycling from splenic macrophages and iron absorption in the duodenum via hepcidin, is also involved in iron deficiency (Cappellini et al., 2020). Hepcidin, a liver‐derived peptide hormone, regulates iron homeostasis through the binding to ferroportin, the only known cellular iron exporter, that blocks its function by occlusion or degradation (Nemeth et al., 2004, Nemeth & Ganz, 2006). As a result, hepcidin hampers iron absorption and iron recycling.

Iron deficiency may be associated with undernutrition (Percy et al., 2017), which is a health issue that has been recognized in young Japanese women. The Japanese National Health Survey reported that 20.7% of young Japanese women had a low body mass index (BMI) < 18.5 kg/m^2^ (Ministry of Health, Labour and Welfare of Japan, 2019). This trend is considered mainly due to an excessive drive for thinness in young women, which may be reinforced by societal culture and media (Holland & Tiggemann, 2016; Smith & Joiner, 2008). Research in anorexia nervosa patients demonstrated significantly higher serum hepcidin levels with normal inflammation levels when compared with controls who were not diagnosed with an eating disorder, providing additional evidence that a severe energy deficiency may exacerbate hepcidin levels in the absence of inflammation (Papillard‐Marechal et al., 2012). Therefore, undernutrition may indirectly affect iron status mediated by augmented hepcidin activity.

Low energy availability (LEA), which may occur as a result of purposefully reduced energy intake (EI) or increases in energy expenditure (EE) and/or failure to increase EI (Areta et al., 2021), also increased hepcidin levels above baseline during endurance training or a simulated military task (Hennigar et al., 2021; Ishibashi et al., 2020). In a recent review, Badenhorst et al. (2019) suggested that LEA appears to elevate hepcidin levels above baseline in the absence of inflammation in athletes or recreationally active individuals via the peroxisome proliferator‐activated receptor‐gamma coactivator 1‐α (PPARGC1α)/cyclic adenosine monophosphate‐responsive element‐binding protein‐H (CREBH) pathway. Activation of that pathway has been shown to stimulate HAMP gene expression, and subsequently increase hepcidin levels (Vecchi et al., 2014).

While the evidence is available from reviews and from patients with medically diagnosed eating disorders, there is limited evidence on the influence of an energy deficit and/or the restriction of key macronutrients (e.g., carbohydrates) that may occur in conjunction with an energy‐deficient diet in females on iron status and hepcidin activity. Acute manipulation of carbohydrate (CHO) availability is thought to be associated with iron metabolism via hepcidin regulation (McKay et al., 2020). Moreover, gluconeogenesis signaling via the PPARGC1α/CREBH pathway (Vecchi et al., 2014). Considering these previous reports, the situation with concomitant decreases in energy and CHO intake may exacerbate hepcidin regulation.

The purpose of this study was to compare the impact of an energy‐deficient diet with low CHO to an energy‐deficient diet with moderate CHO during three consecutive days on the hepcidin response, hematological variables, and energy metabolism in young Japanese women.

## 2 MATERIALS AND METHODS

### 2.1 Participants

Twenty‐two healthy females participated in this study. Participants are required to meet the following criteria: (i) University and graduate students between 18 and 24 yrs of age; (ii) no history of using oral contraceptive pills; (iii) having regular menstrual cycle (menstrual cycles from 21 to 35 days in length [Elliott‐Sale et al., 2021]); (iv) non‐pregnant; (v) non‐smokers; (vi) subject symptoms of anemia and/or not diagnosed anemia. All participants were informed of the purpose, experimental overview, and the potential risks involved with the present study. Subsequently, written informed consent was obtained. The present study was conducted after being approved by the Ethical Committee of Human Experiments at Ritsumeikan University (BKC_2020_009), in accordance with the Declaration of Helsinki.

They were randomly assigned to either an energy‐deficient diet with low CHO intake (ED + LCHO: *n* = 11, 22%PRO, 39%FAT, 39%CHO, 1122 kcal) or an energy‐deficient diet with moderate CHO intake (ED: *n* = 11, 18%PRO, 19%FAT, 63%CHO, 1162 kcal). Sixteen participants who completed the intervention were included in the data analysis (mean ± standard deviation (SD), age: 21.3 ± 1.2 years, height: 161.3 ± 4.9 cm, body weight (BW): 55.3 ± 6.2 kg, BMI: 21.2 ± 1.5 kg/m^2^). Five participants presented with severe iron deficiency (serum ferritin < 12 ng/mL, Asakura et al., 2009), and a participant who presented inflammation due to infection (plasma IL‐6 > 4 pg/mL with subjective symptom) were excluded from the data analysis.

### 2.2 Experimental overview

Participants visited the laboratory twice throughout the experiment. On the first visit (8:00–9:00 on Day 1), they completed a series of measurements before the dietary intervention, which included blood sample collection, resting respiratory sample collection, evaluation of body composition, and subjective variables. They then commenced a restricted dietary intake intervention period for three consecutive days (Day 1, Day 2, and Day 3). During the intervention period, participants consumed only the prescribed diet (3 days, eating three times a day), and maintained their habitual physical activity levels. Water intake was allowed ad libitum. Diets were provided as pre‐packaged food (Nissin Healthcare Food Service Co., Ltd.) to take home and participants were instructed to eat the provided food in their own time throughout the day while aiming to finish all the provided food at 22:00 the day before the post‐measurement. Table 1 shows the structure and provision of diets ensured that total energy intake and percentage of macronutrients. The diet's macro and micronutrient content were calculated using dedicated software (Excel Eiyo‐kun FFQg version 8.0; Kenpaku‐sha). The study participants had high compliance with the dietary intervention. All participants consumed all food provided for both interventions. On the second visit, at the completion of the 3‐day dietary intervention (8:00–9:00 on Day 4), baseline measurements were repeated in the morning following completion of the intervention period. All measurements before and after the intervention period were conducted following an overnight fast (>10 h). The phases of the menstrual cycle were estimated by self‐reported the schedule for their menstrual cycle ~2 months prior to commencement of this study (calendar‐based counting) and analyzing the hormonal concentration in pre‐and post‐measurements, but ovulation was not monitored.

**TABLE 1:** Total energy intake and macronutrients during the intervention period

Variables		ED + LCHO				ED			
		Day1	Day2	Day3	Average	Day1	Day2	Day3	Average
Energy intake	(kcal)	1097	1194	1076	1122	1159	1183	1143	1162
Carbohydrate	(g)	112.0	110.0	106.6	109.6	184.0	184.9	182.4	183.8
	(g/kg)	2.1 ± 0.3*	2.0 ± 0.3*	2.0 ± 0.3*	2.0 ± 0.3*	3.4 ± 0.3	3.4 ± 0.3	3.4 ± 0.3	3.4 ± 0.3
	(for energy)	41%	37%	40%	39%	64%	63%	64%	63%
Protein	(g)	58.8	62.9	64.8	62.2	50.6	50.3	52.7	51.2
	(g/kg)	1.1 ± 0.1*	1.2 ± 0.2*	1.2 ± 0.2*	1.2 ± 0.2*	0.9 ± 0.1	0.9 ± 0.1	1.0 ± 0.1	0.9 ± 0.1
	(for energy)	21%	21%	24%	22%	17%	17%	18%	18%
Fat	(g)	46.0	55.8	43.4	44.3	24.5	26.9	22.5	24.6
	(g/kg)	0.9 ± 0.1*	1.0 ± 0.1*	0.8 ± 0.1*	0.9 ± 0.1*	0.5 ± 0.0	0.5 ± 0.0	0.4 ± 0.0	0.5 ± 0.0
	(for energy)	38%	42%	36%	39%	19%	20%	18%	19%
Iron	(mg)	7.9	9.4	8.9	8.7	14.8	5.1	6.8	8.9

*Note*: Values are means ± SD.

Abbreviations: ED + LCHO, a group of energy deficient with a low carbohydrate diet; ED, a group of energy deficient diet.

* Significant different (*p* < 0.05) to ED by an independent *t*‐test.

### 2.3 Blood variables

Before and after the intervention period, fasting (>10 h following a previous meal) blood samples were collected in a supine position using a serum separating tube (for serum), Na2‐ and K2‐ethylenediaminetetraacetic acid (EDTA) containing tubes (for plasma and whole blood, respectively). Blood glucose level was measured immediately after blood collection using the remaining blood in the needle by an automatic blood glucose analyzer (Free Style; Nipro Corporation, Osaka, Japan). Serum separating tubes and Na2‐EDTA containing tubes were centrifuged at 1710 *g* and 4°C for 10 min to obtain serum and plasma. These samples were frozen at −60°C until analysis. Whole blood samples were stored in the refrigerator and these samples were sent to a clinical laboratory (SRL Inc.) for further analysis. Serum iron, ferritin, haptoglobin, total ketone bodies (acetoacetic acid and β‐hydroxybutyric acid), estrogen, and progesterone were measured from an obtained serum sample, and complete blood count [red blood cell (RBC), white blood cell (WBC), hemoglobin (Hb), hematocrit (Htc), platelet, mean corpuscular hemoglobin (MCH), mean corpuscular hemoglobin concentration (MCHC)] and reticulocyte from whole blood sample were measured in a clinical laboratory (SRL Inc.). Changes in plasma volume (ΔPV) was calculated using the equation based on Hb and Hct (Dill & Costill, 1974). Serum lipid hydroperoxide (LPO) was evaluated in the different clinical laboratories (Japan Institute for the Control of Aging, NIKKEN SEIL Co., Ltd.). Serum leptin, hepcidin, and plasma IL‐6 levels were analyzed using enzyme‐linked immunosorbent assay (ELISA) kits (R&D systems). The coefficients of variations (CV) were 3.9% (leptin), 5.9% (hepcidin), 1.9% (IL‐6), 3.8% (estradiol), 4.1% (progesterone), 4.1% (ferritin), 5.1% (haptoglobin) and 5.0% (total ketone bodies).

### 2.4 Body composition

Participants arrived at the laboratory from 8:00 am to 8:30 am after an overnight fast, and they subsequently measured their body composition pre and post‐intervention. Bodyweight (BW), fat‐free mass (FFM), fat mass (FM), and total body water were measured by bioimpedance analysis using a body composition analyzer (InBody 770, In Body Japan Inc.).

### 2.5 Respiratory variables

Resting expired gas samples were collected pre‐ and post‐intervention from 8:15 am to 9:00 am after measuring body composition. Participants rested on a reclining bed during this measurement. The samples were collected in a supine position for 10 min after 10 min rest. Participants wore a face mask during collecting the expired gas sample by breath‐by‐breath method, and oxygen uptake (VO_2_), carbon dioxide output (VCO_2_), and respiratory exchange ratio (RER) were evaluated using an automatic gas analyzer (AE300S; Minato Medical Science). The calibrations for O_2_ and CO_2_ sensors were performed using a gas containing known O_2_ and CO_2_ concentrations. The volume transducer was calibrated with a 2 L syringe. Resting metabolic rate (RMR), and the amount of CHO oxidation and fat oxidation were calculated using VO_2_ and VCO_2_ values (Jeukendrup & Wallis, 2005; Weir, 1949).

$\begin{array}{c} \mathrm{RMR}\left(\text{kcal}/\min\right)=3.941\times {\mathrm{VO}}_{2}\left(L/\min\right)+1.106\\ \times {\mathrm{VCO}}_{2}\left(L/\min\right). \end{array}$

$\begin{array}{c} \mathrm{CHO}\text{oxidation}\left(g/\min\right)=4.585\times {\mathrm{VCO}}_{2}\left(L/\min\right)\\ –3.226\times {\mathrm{VO}}_{2}\left(L/\min\right). \end{array}$

$\begin{array}{c} \mathrm{Fat}\text{oxidation}\left(g/\min\right)=1.695\times {\mathrm{VO}}_{2}\left(L/\min\right)\\ –1.701\times {\mathrm{VCO}}_{2}\left(L/\min\right). \end{array}$

### 2.6 Subjective variables

Before and after the intervention period, participants answered a subjective survey that assessed their feelings of hunger, sleepiness, fatigue, heaviness, and discomfort using 1–5 scales (1 = little, 5 = a lot). This questionnaire was based on Profile of Mode States (POMS, McNair et al., 2003).

### 2.7 Physical activities during the intervention period

During the three consecutive days of the intervention period, the average number of steps and average daily EE were evaluated by all participants wearing an accelerometer (Actimarker, Panasonic Corporation) during the day except for during sleep and when taking a bath.

### 2.8 Statistical analysis

Statistical analysis was performed using JASP version 0.16.2 (JASP Team, 2020). A separate two‐way analysis of variance (ANOVA) with repeated measures was used to analyze the time main effect (pre, post), group main effect (ED + LCHO, ED), and interaction between these two factors. When a significant interaction was observed, Bonferroni adjustments were applied for post‐hoc comparison. Average daily EE, number of steps, and relative changes in hepcidin, leptin, and IL‐6 level levels were compared between the two groups using an independent *t*‐test. Pearson correlation coefficient was used to determine the relationship between ferritin, iron, hepcidin, estradiol, progesterone, ketone bodies, and the relative change from pre to post. Effect sizes (ES) are summarized as partial eta squared (*η*_*p*_^2^) for ANOVA and as Cohen's d for post hoc, Bonferroni and *t*‐test. ES of *d* = 0.2 is classified as “small”, *d* = 0.5 is as “medium”, *d* = 0.8 is “large (Cohen, 1988). All data are presented as means ± SD and the statistical significance level was accepted at *p* < 0.05.

## 3 RESULTS

### 3.1 Physical activity level and body composition

Average daily EE during the intervention period did not differ significantly between the two groups (1926 ± 193 kcal/day in ED + LCHO, 1833 ± 192 kcal/day in ED, *p* = 0.352, *d* = 0.482; 95% CI [−0.522, 1.469]). Number of steps per day did not differ between the two groups (10,165 ± 2696 steps/day in ED + LCHO, 10258 ± 3604 steps/day in ED, *p* = 0.954, *d* = 0.029; 95% CI [−1.009, 0.951]). Energy balance, subtracting EE from EI, was similar between the groups (−804 ± 193 kcal in ED + LCHO, −671 ± 192 kcal in ED, *p* = 0.190, *d* = 0.690; 95% CI [−0.334, 1.690]). Energy deficit that EI divided by EE and multiplied by 100 was also similar between groups (58.7 ± 5.4% in ED + LCHO, 64.1 ± 7.3% in ED, *p* = 0.120, *d* = −0.829; 95% CI [−1.842, 0.211]). BW showed significant reduction after the intervention (*p* < 0.001, *d* = 0.115; 95% CI [0.052, 0.179]); Table 2. FM showed significant reduction after the intervention (*p* < 0.001, *d* = 0.187 95% CI [0.094, 0.281]; Table 2. No significant changes over the intervention were found for FFM and TBW; Table 2.

**TABLE 2:** Body compositions

Variables	ED + LCHO		ED		ANOVA *p* value		
					Effect size (*η*_*p*_^2^)		
	Pre	Post	Pre	Post	Interaction	Time	Group
Body weight (kg)	57.1 ± 6.7	56.3 ± 6.7	53.5 ± 5.5	52.8 ± 5.5	0.879	<0.001***	0.261
					(0.002)	(0.708)	(0.089)
Fat‐free mass (kg)	41.4 ± 4.6	41.4 ± 4.4	40.1 ± 3.3	40.0 ± 3.3	0.610	0.560	0.473
					(0.019)	(0.025)	(0.037)
Fat mass (kg)	15.6 ± 3.9	14.9 ± 3.8	13.4 ± 2.5	12.9 ± 2.5	0.211	<0.001***	0.208
					(0.110)	(0.800)	(0.111)
Total body water (L)	30.3 ± 3.4	30.3 ± 3.2	29.3 ± 2.4	29.1 ± 2.4	0.593	0.468	0.456
					(0.021)	(0.038)	(0.040)

*Note*: Values are means ± SD.

Abbreviations: ED + LCHO, a group of energy deficient with a low carbohydrate diet; ED, a group of energy deficient diet; *η*_*p*_^2^, partial eta squared.

*** *p* < 0.01.

### 3.2 Scores of subjective variables

Hunger score presented significant main time effect (*p* < 0.001, *η*_p_^2^ = 0.620), without no significant interaction (*p* = 0.060, *η*_*p*_^2^ = 0.231) and main group effect (*p* = 0.285, *η*_*p*_^2^ = 0.081). Hunger score significantly increased after intervention (*p* < 0.001, *d* = −1.140; 95% CI [−1.807, −0.473]). Heaviness score presented significant main time effect (*p* = 0.040, *η*_*p*_^2^ = 0.268), without no significant interaction (*p* = 0.128, *η*_*p*_^2^ = 0.157) and main group effect (*p* = 0.499, *η*_*p*_^2^ = 0.033). Heaviness score significantly increased after intervention (*p* = 0.040, *d* = −0.447; 95% CI [−0.896, 0.003]). Discomfort score presented significant main time effect (*p* = 0.010, *η*_*p*_^2^ = 0.391), without no significant interaction (*p* = 0.334, *η*_*p*_^2^ = 0.067) and main group effect (*p* = 0.428, *η*_*p*_^2^ = 0.045). Discomfort score significantly increased after the intervention (*p* = 0.010, *d* = −0.567; 95% CI [−1.022, −0.111]). Sleepiness score and fatigue score did not change over the intervention (*p* = 0.471, *η*_*p*_^2^ = 0.038; *p* = 0.179, *η*_*p*_^2^ = 0.125).

### 3.3 Hematological variables and iron status

WBC decreased after the intervention (*p* = 0.001, *d* = 0.783; 95% CI [0.270, 1.297]); Table 3. RBC decreased after the intervention (*p* = 0.026, *d* = 0.454; 95% CI [0.0030, 0.877]). Significant difference in RBC was observed between the groups (*p* = 0.047, *d* = 1.012, 95% CI [−0.068, 2.091]). Hb level decreased after the intervention (*p* = 0.003, *d* = 0.575; 95% CI [0.169, 0.981]). Hct levels significantly decreased after the intervention (*p* = 0.001, *d* = 0.764; 95% CI [0.268, 1.260]. Significant difference in Hct was observed between the groups (*p* = 0.044, *d* = 1.026, 95% CI [−0.052, 2.103]). ΔPV did not differ between the groups from pre to post intervention (+4.74 ± 5.90% in ED + LCHO, +6.27 ± 5.26% in ED, *p* = 0.593, *d* = −0.274; 95% CI [−1.254, 0.716]). Reticulocyte levels showed a trend toward significant main time effect. Serum ferritin levels increased after the intervention (*p* = 0.003, *d* = −0.711; 95% CI [−1.213, −0.208]). Serum iron levels showed a trend toward significant interaction; significantly increased after the intervention (*p* < 0.001, *d* = −1.171; 95% CI [−1.861, −0.481]). No significant differences in serum ferritin for the relative changes from pre to post (60.2 ± 47.8% in ED + LCHO, 52.6 ± 45.8% in ED, *p* = 0.753, *d* = 0.160; 95% CI [−0.824, 1.139]) and iron (45.6 ± 36.0% in ED + LCHO, 20.4 ± 21.1% in ED, *p* = 0.110, *d* = 0.854; 95% CI [−0.189, 1.869]) were found. Serum TIBC did not show any interaction or main effect. A significant difference in serum haptoglobin was observed between the groups (*p* = 0.019, *d* = −1.197; 95% CI [−2.283, −0.112]).

**TABLE 3:** Hematological, hormonal and inflammatory variables

Variables	ED + LCHO		ED		ANOVA *p* value		
					Effect size (*η*_*p*_^2^)		
	Pre	Post	Pre	Post	Interaction	Time	Group
WBC (/μL)	5775 ± 1058	4988 ± 544	5088 ± 843	4525 ± 921	0.516	0.001***	0.169
					(0.031)	(0.534)	(0.130)
RBC (×10^4^/μL)	455 ± 28	440 ± 22	424 ± 30	413 ± 34	0.657	0.026***	0.047**
					(0.015)	(0.306)	(0.252)
Hb (g/dL)	13.6 ± 0.7	13.3 ± 0.4	13.1 ± 0.9	12.7 ± 0.7	0.668	0.003***	0.136
					(0.014)	(0.483)	(0.151)
Hct (%)	40.4 ± 1.7	39.3 ± 0.8	38.8 ± 2.1	37.3 ± 1.9	0.545	0.001***	0.044*
					(0.027)	(0.543)	(0.259)
MCV (fL)	88.9 ± 4.3	89.4 ± 3.9	91.7 ± 3.3	90.5 ± 4.1	0.060*	0.465	0.318
					(0.230)	(0.039)	(0.071)
MCH (pg)	30.0 ± 1.2	30.2 ± 0.9	29.8 ± 3.7	30.7 ± 1.4	0.572	0.355	0.841
					(0.023)	(0.061)	(0.003)
MCHC (%)	33.8 ± 0.8	33.8 ± 0.6	33.8 ± 0.7	34.0 ± 0.5	0.576	0.448	0.658
					(0.023)	(0.042)	(0.014)
Platelets (×10^4^/μL)	25.7 ± 4.7	23.6 ± 4.2	22.6 ± 3.3	22.4 ± 2.9	0.169	0.129	0.248
					(0.131)	(0.157)	(0.094)
Reticulocyte (‰)	11.6 ± 2.9	10.0 ± 3.3	11.4 ± 1.9	9.9 ± 1.8	0.933	0.051*	0.862
					(0.001)	(0.246)	(0.002)
Ferritin (ng/mL)	30.4 ± 19.6	43.4 ± 18.6	28.2 ± 13.5	45.1 ± 29.1	0.654	0.003***	0.982
					(0.015)	(0.477)	(0.000)
Iron (μg/dL)	91 ± 21	126 ± 16	88 ± 26	103 ± 20	0.065*	< 0.001***	0.189
					(0.223)	(0.613)	(0.120)
TIBC (μg/dL)	333 ± 31	320 ± 37	315 ± 43	313 ± 30	0.483	0.348	0.464
					(0.036)	(0.063)	(0.039)
TSAT (%)	27 ± 5	40 ± 7^a^	28 ± 8	33 ± 5	0.027*	< 0.001***	0.278
					(0.303)	(0.663)	(0.083)
Haptoglobin (mg/mL)	51 ± 35	34 ± 22	83 ± 39	84 ± 36	0.235	0.297	0.019**
					(0.099)	(0.077)	(0.333)
Hepcidin (ng/mL)	13.1 ± 11.8	29.9 ± 13.5	13.0 ± 8.8	19.9 ± 12.5	0.124	0.002***	0.336
					(0.161)	(0.525)	(0.066)
Leptin (ng/mL)	12.5 ± 6.9	6.2 ± 3.3^a^	7.2 ± 3.1	5.0 ± 1.4	0.031**	< 0.001***	0.107
					(0.291)	(0.628)	(0.175)
IL‐6 (pg/dL)	0.84 ± 0.40	0.66 ± 0.18	1.09 ± 0.64	0.93 ± 0.54	0.867	0.077*	0.252
					(0.002)	(0.207)	(0.092)
LPO (mmol/mL)	3.2 ± 0.3	3.2 ± 0.3	3.6 ± 0.7	3.3 ± 0.6	0.472	0.312	0.261
					(0.038)	(0.073)	(0.089)

*Note*: Values are means ± SD.

Abbreviations: ED + LCHO, a group of energy deficient with low carbohydrate diet; ED, a group of energy deficient diet; TIBC, total iron binding capacity; TSAT, transferrin saturation; IL‐6, interleukin‐6; LPO, lipid peroxides; *η*_*p*_^2^, partial eta squared.

^a^ Significant difference with pre by Bonferroni post hoc.

* *p* < 0.1

** *p* < 0.05

*** *p* < 0.01.

### 3.4 Hormonal and inflammatory variables

**FIGURE 1:**
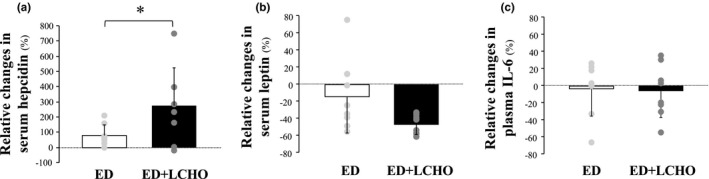
Relative changes in serum hepcidin (a), leptin (b), and plasma IL‐6 (c) from baseline values. Values are means ± SD. ED + LCHO, a group of energy deficient with a low carbohydrate diet; ED, a group of energy deficient diet. *Significant difference between the groups by an independent *t*‐test.

Serum hepcidin levels significantly increased after the intervention (*p* = 0.022, *d* = −1.009; 95% CI [−1.670, −0.347]); Table 3. Relative change from pre to post in serum hepcidin level was significantly higher in ED + LCHO (264.3 ± 246.8%) than in ED group (68.9 ± 62.4%, *p* = 0.048, *d* = 1.086; 95% CI [0.011, 2.127]); Figure 1. Serum leptin level significantly decreased after the intervention in ED + LCHO (*p* < 0.001, *d* = 1.518, 95% CI [0.310, 2.727]), whereas it did not change in ED (*p* = 0.622, *d* = 0.515; 95% CI [−0.398, 1.428]; Table 3. Relative change from pre to post in serum leptin levels did not differ between the groups (−49.7 ± 11.1% in ED + LCHO, −18.1 ± 47.3% in ED, *p* = 0.087, *d* = −0.921; 95% CI [−1.943, 0.130]); Figure 1. The relative change in leptin levels did not correlate significantly with the relative change in hepcidin levels (*p* = 0.274, *r* = −0.291; 95% CI [−0.688, 0.239]). Plasma IL‐6 levels showed a trend toward main time effect; however, these levels did not show any significant interaction or main effect. Relative changes from pre to post did not differ significantly between the groups (−10.3 ± 32.1% in ED + LCHO, −9.8 ± 29.0% in ED, *p* = 0.977, *d* = −0.015; 95% CI [−0.994, 0.966]); Figure 1.

### 3.5 Relationship between hepcidin response and iron‐related variables

A significant positive correlation was observed between serum ferritin and hepcidin levels (pre, *r* = 0.636, 95% CI [0.204, 0.860], *p* = 0.008; post, *r* = 0.528, 95% CI [0.043, 0.811], *p* = 0.036). A significant positive correlation was observed between serum iron and serum hepcidin levels (pre, *p* = 0.027, *r* = 0.549, 95% CI [0.074, 0.821]; post, *p* < 0.001, *r* = 0.787, 95% CI [0.477, 0.923]).

### 3.6 Blood glucose and serum ketone bodies levels

**FIGURE 2:**
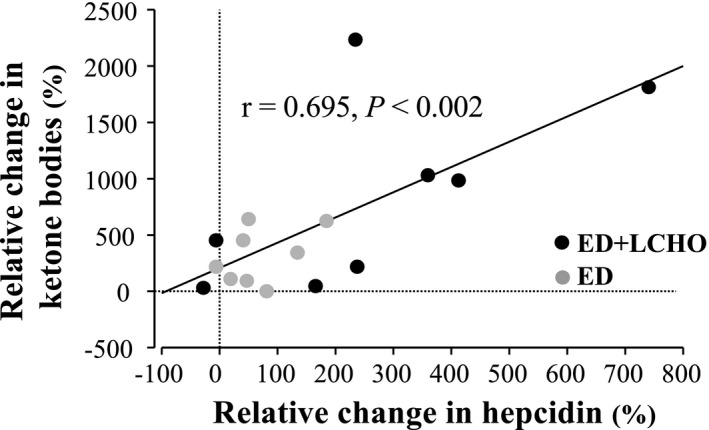
Relationship between relative changes in serum hepcidin and total ketone bodies levels from baseline values. ED + LCHO, a group of energy deficient with a low carbohydrate diet; ED, a group of energy deficient diet.

Blood glucose level decreased after the intervention (*p* < 0.001, *d* = 1.212; 95% CI [−0.461, 1.962]; Table 4). Serum total ketone bodies levels increased after intervention in ED + LCHO group (*p* < 0.001; *d* = −1.901, 95% CI [−3.263, −0.539]); however, these levels did not change in ED (*p* = 0.128; *d* = −0.763, 95% CI [−1.724., 0.198]). Postintervention serum total ketone bodies levels were significantly higher in ED + LCHO than in ED (*p* = 0.025; *d* = 1.624, 95% CI [−0.097, 3.345]). Serum acetoacetic acid levels increased after the intervention (*p* < 0.001, *d* = −1.131; 95% CI [−1.775, −0.487]). ED + LCHO presented higher level of serum acetoacetic acid compared with ED (+46.125, *p* = 0.049; *d* = 0.964, 95% CI [−0.072, 2.000]). Serum β‐hydroxybutyric acid levels increased after intervention in ED + LCHO group (*p* < 0.001, *d* = −2.014, 95% CI [−3.427, −0.602]); however, these levels did not change in ED (*p* = 0.157; *d* = −0.773, 95% CI [−1.688, 0.222]). Postintervention serum β‐hydroxybutyric acid level was significantly higher in ED + LCHO than in ED (*p* = 0.017; *d* = 1.711, 95% CI [−0.036, 3.457). Relative change in serum hepcidin level correlated with total serum ketone bodies (*p* = 0.003; *r* = 0.695, 95% CI [0.304, 0.886]), acetoacetic acid (*p* = 0.038; *r* = 0.521, 95% CI [0.034, 0.808]), and β‐hydroxybutyric acid levels (*p* = 0.001; *r* = 0.739, 95% CI [0.385, 0.904]) in both ED + LCHO and ED groups; Figure 2.

**TABLE 4:** Blood glucose and serum ketone bodies levels

Variables	ED + LCHO		ED		ANOVA *p* value		
					Effect size (*η*_*p*_^2^)		
	Pre	Post	Pre	Post	Interaction	Time	Group
Glucose (mg/dL)	87 ± 7	76 ± 5	89 ± 6	84 ± 8	0.163	<0.001***	0.111
					(0.134)	(0.567)	(0.171)
Total ketone bodies (μmol/L)	195 ± 293	628 ± 291^a^ ^,^ ^b^	84 ± 82	258 ± 174^a^	0.016**	<0.001***	0.036**
					(0.348)	(0.745)	(0.278)
Acetoacetic acid (μmol/L)	53 ± 72	120 ± 47	20 ± 17	61 ± 38	0.232	<0.001***	0.049**
					(0.100)	(0.646)	(0.249)
β‐hydroxybutyric acid (μmol/L)	142 ± 221	507 ± 24^a^ ^,^ ^b^	64 ± 65	197 ± 137^a^	0.008***	<0.001***	0.034**
					(0.403)	(0.756)	(0.284)

*Note*: Values are means ± SD. Values are means ± SD.

Abbreviations: ED + LCHO, a group of energy deficient with low carbohydrate diet; ED, a group of energy deficient diet; ηp2, partial eta squared.

^a^ Significant difference with pre by Bonferroni post hoc.

^b^ Significant difference with ED by Bonferroni post hoc.

** *p* < 0.05

*** *p* < 0.01.

### 3.7 Resting metabolic rate and substrate oxidation

RMR did not present any interaction or main effect (0.864 ± 0.092 to 0.885 ± 0.070 kcal/min in ED + LCHO, 0.863 ± 0.079 to 0.852 ± 0.065 kcal/min in ED). RER presented only main time effect (*p* < 0.001, *η*_*p*_^2^ = 0.763); significantly decreased after the intervention (0.83 ± 0.06 to 0.76 ± 0.04 in ED + LCHO, 0.83 ± 0.05 to 0.75 ± 0.06 in ED; *p* < 0.001, *d* = 1.444; 95% CI [0.720, 2.169]). CHO oxidation presented only main time effect (*p* < 0.001, *η*_*p*_^2^ = 0.753); significantly decreased after the intervention (0.097 ± 0.041 to 0.039 ± 0.040 g/min in ED + LCHO, 0.106 ± 0.047 to 0.042 ± 0.041 mg/min in ED, *p* < 0.001, *d* = 1.438; 95% CI [0.709, 2.167]). Fat oxidation presented only main time effect (*p* < 0.001, *η*_*p*_^2^ = 0.732); significantly increased after the intervention (0.052 ± 0.022 to 0.078 ± 0.018 g/min in ED + LCHO, 0.049 ± 0.019 to 0.073 ± 0.015 mg/min in ED, *p* < 0.001, *d* = −1.353; 95% CI [−2.054, −0.651]).

### 3.8 Reproductive hormones

ED + LCHO group included three participants who were in the follicular phase (during menses or early follicular phase) and five participants who were in the luteal phase, while the ED group included five participants who were in the follicular phase and three participants who were in the luteal phase. Serum estradiol level did not correlate significantly with neither serum iron (*p* = 0.643, *r* = 0.126, 95% CI [−0.395, 0.585]), serum ferritin (*p* = 0.508, *r* = 0.178, 95% CI [−0.348, 0.619]) or serum hepcidin levels (*p* = 0.951, *r* = −0.017, 95% CI [−0.508, 0.483]). Serum progesterone level did not correlate with neither serum ferritin (*p* = 0.683, *r* = −0.111, 95% CI [−0.575, 0.407]), serum iron (*p* = 0.553, *r* = −0.160, 95% CI [−0.608, 0.364]) or serum hepcidin levels (*p* = 0.236, *r* = −0.314, 95% CI [−0.701, 0.215]).

## 4 DISCUSSION

The present study investigated the influence of three consecutive days of a reduced EI with either moderate CHO intake or a low CHO intake (energy deficient and low CHO availability status) on hepcidin activity, hematological variables, and energy metabolism in young Japanese women. The main findings of the present study with regards to iron regulation were that an energy‐deficient diet increased fasting hepcidin levels without any observable changes in inflammation. Moreover, the relative increase in fasting hepcidin levels following the intervention was greater when energy‐deficient was combined with low CHO intake.

The increases in fasting hepcidin levels in both groups were evident after 3 days of an acute energy‐deficient dietary manipulation, which is consistent with previous observations in research investigating LEA in athletes (Ishibashi et al., 2020), negative energy balance during the simulated military tasks (Hennigar et al., 2021), 18, 42 and 66‐h of fasting state (Troutt et al., 2012), and in clinical research with anorexia nervosa patients (Papillard‐Marechal et al., 2012). Energy‐deficient in the present study was equivalent to or greater than that in Hennigar et al. (2021), however, the present study showed a greater %change in hepcidin levels after the intervention compared to the previous study. The present study did not utilize the structured exercise session, and the specific experimental design was the pivotal difference from the above previous studies. IL‐6, a marker of inflammation, upregulates hepcidin through the Janus kinase (JAK) 2/ signal transducer and activator of transcription (STAT) 3 signaling pathway (Wrighting & Andrews, 2006). In the present study, no detectable increase in IL‐6 was observed during the three‐days intervention, and this finding appears to align with previous research on extremely severe energy‐deficient (i.e., fasting). Vecchi et al. (2014) observed that gene expressions of IL‐6, tumor necrosis factor (TNF) or C‐reactive protein (CRP) did not change significantly following 24 and 48 h of fasting in mice, while gene expression of serum hepcidin significantly increased in mice. Moreover, the effect of erythropoiesis should be taken into consideration for interpreting of hepcidin response, when EE was increased with exercise training (Moretti et al., 2018). Therefore, it is worth noting that the mechanism promoting hepcidin elevations in the present study (i.e., energy deficient‐induced hepcidin elevation) would be different from that underlying exercise‐induced hepcidin elevations, proposed to be due to increases in IL‐6 post‐exercise (Banzet et al., 2012).

The relative increase in hepcidin levels over 3 days of the dietary intervention was approximately three times higher in the ED + LCHO group. It is possible that augmented ketone bodies levels may be a factor for increased hepcidin levels in the ED + LCHO group through activation of the PPARGC1α/CREBH pathway (Vecchi et al., 2014), which is supported by the correlation between %change in serum ketone bodies and hepcidin levels in the present study. Starvation is suggested to promote glycogenolysis and glucose synthesis, subsequently activating gluconeogenesis to provide glucose to other tissues (Han et al., 2016). Additionally, caloric restriction, carbohydrate restriction, or fasting state increase β‐oxidation in the liver and gluconeogenesis while decreasing acetyl‐CoA oxidation in the TCA cycle, which causes increased ketone synthesis (Browning et al., 2008, 2012). Increased β‐oxidation, ketone bodies, and gluconeogenesis will activate PPARGC1α (Lin et al., 2005) that stabilize the CREBH proteins (Vecchi et al., 2014). Therefore, elevated hepcidin from pre‐ to post‐intervention would be indirectly associated with increases in serum ketone bodies, consequently, the ED + LCHO group presented higher hepcidin increase compared to the ED group. In an animal study, rats with 30% less diet than normal food intake demonstrated a significant decrease in liver glycogen content after a 5 day‐intervention when compared to a trial with feeding ad libitum (Ugochukwu & Figgers, 2006). In addition, rats with 20% or 30% less diet than normal food intake decreased hepatic weight and enhanced lipid metabolism further suggesting a decrease of liver glycogen content as proposed by these authors (Kobayashi et al., 2021). Therefore, the energy deficiency plus the low CHO intake in our participants may have lowered liver glycogen content suggested by lower blood glucose and higher ketone body levels (Gonzalez et al., 2016; Han et al., 2016). We speculate that liver glycogen plays an important role in hepcidin regulation in situations of energy deficit or/and CHO restriction. Although, further research is required, liver glycogen content might be a key factor regulating hepcidin production in energy‐deficient conditions.

Significant elevations of serum ferritin and iron were observed in both groups. A phenomenon of increased serum ferritin level was observed in previous research which is occurred energy deficiency (Hennigar et al.,  2021; Ishibashi et al., 2020; Troutt et al., 2012). Troutt et al. (2012) showed that resting hepcidin level was significantly increased following 18, 42 and 66 h of fasting, which is proposed to be the result of attenuated erythropoiesis and sequestering of iron to maintain iron in tissues during a fasted state. Even though the magnitude of energy‐deficiency is different level compared to Troutt et al. (2012), it is possible that an energy‐deficient diet with subsequent augmented hepcidin and serum ferritin may result in impaired iron metabolism. Increased serum iron levels may have facilitated an increase in hepcidin levels due to hepcidin production in hepatocytes being regulated by intracellular and extracellular iron concentrations (Nemeth & Ganz, 2006). This may be supported by the tendency of a positive correlation between the relative changes in serum iron and hepcidin levels. However, the mechanism underlying increased serum iron levels is unclear. We can rule out the influence of dietary intake or hemolysis on increased serum iron levels due to our samples being collected in the morning following a >10 h overnight fast and in a rested state. As a support of this notion, markers of hemolysis (i.e., haptoglobin and LPO) did not change throughout the trial in either group (Lippi & Sanchis‐Gomar, 2019). Generally, ~20–25 mg Fe/day is required for turnover of RBCs, and iron from recycled senescent RBCs is utilized to maintain that cycle (Sangkhae & Nemeth, 2017). Turnover of RBCs may have failed to function properly due to increased hepcidin concentration after the acute intervention (Sangkhae & Nemeth, 2017), as the result, serum iron could have presented higher value.

A significant reduction in WBCs after the intervention was consistent with the previous studies which have demonstrated a decrease in BW due to anorexia (De Filippo et al., 2016). Additionally, RBCs, Hb, and Hct showed a significant decreases and reticulocytes showed a tendency of decrease. These results may be due to the acute energy‐deficient implemented in both groups. Previous studies have reported that decreased food intake may cause hemoconcentration due to decreases in water intake (Kobayashi et al., 2021). Contrary to the previous study, a decrease in PV was not observed in the present intervention in either of the two groups, that is hemoconcentration did not occur in the present study (Dill & Costill, 1974). PV expansion has previously been seen after the training session with heat stress (Racinais et al., 2015) or high CHO and protein intake (Okazaki et al., 2009), however, again the present study did not incorporate any of the aforementioned situations. Thus, the influence on PV and the ensuing effects on hematological markers in the present study are minimal. Decreased hematological variables in energy‐deficient status may have been caused by the acute suppression of hematopoiesis (Mountjoy et al., 2018).

The present study has several limitations. Estradiol has been shown to attenuate hepcidin expression in vitro studies (Hou et al., 2012; Yang et al., 2012). Fluctuations of serum iron and hepcidin during the menstrual cycle have been reported by Angeli et al. (2016) and Lainé et al. (2016), indicating that serum hepcidin and iron will tend to follow a set pattern, with lower levels in the early follicular phase, that then gradually increase in later follicular phase and rebound in luteal phase. The ED + LCHO group did have greater number of participants in luteal phase, and that may have resulted in the higher serum hepcidin changes and serum iron levels in this group. The present study has relatively small sample size of participants in each phase of the menstrual cycle and large standard deviations in hormonal and iron status between and within the groups. Therefore, the day‐to‐day variation of hormones on hepcidin response should be taken into consideration when interpretating the present results. As a follow‐up analysis, we examined the relative hepcidin changes in different menstrual phases and intervention groups, consequently, the ED + LCHO group presented higher serum hepcidin levels in both the follicular and luteal phases (336.1 ± 89.1%, 221.3 ± 310.5%, respectively) compared to ED group (79.2 ± 64.5%, 38.1 ± 62.2%, respectively). This may be considered in the interpretation of the results presented.

The relative changes in leptin level presented were lower in the ED + LCHO group when compared to the ED group, albeit not significant. In addition, the ED group had large deviations of leptin changes among the participants. Leptin is a hormone that responds to energy homeostasis (Park & Ahima, 2015). While we induced a comparable negative energy balance that was matched between the groups during the intervention period, it would have been beneficial to consider their normal EI before the intervention. The difference in EI from normal daily intake may have influenced the leptin changes between the groups. Leptin has been identified as an up‐regulators of hepcidin through JAK/STAT pathway (Chung et al., 2007; Falzacappa et al., 2006). However, we observed decreased serum leptin levels, which were not consistent or associated with increased hepcidin levels. This is supported by the lack of correlation between the relative change in leptin levels and the change in hepcidin levels.

In conclusion, three consecutive days of an energy‐deficient diet augmented fasting hepcidin levels in healthy women. Moreover, hepcidin levels in an energy‐deficient diet in addition to low CHO intake were further exacerbated after a 3‐day intervention. These results suggest that energy‐deficit with concomitant reductions in CHO intake may further increase the risk of iron deficiency as a result of greater relative increases in hepcidin levels. There were several limitations in the present study, therefore, further research is required to clear the influence of CHO intake on hepcidin regulation in exercising women as well as non‐exercising women.

### AUTHOR CONTRIBUTION

This study was designed by Nanako Hayashi, Aya Ishibashi, and Kazushige Goto. Data was collected by Nanako Hayashi, Ayame Iwata, and Haruka Yatsutani, and analyzed by Nanako Hayashi, Kazushige Goto. Interpretation of data and manuscript preparation was undertaken by Nanako Hayashi, Claire Badenhorst, and Kazushige Goto.

## FUNDING STATEMENT

This research was funded by Urakami Foundation for Food and Food Culture Promotion.

## CONFLICT OF INTEREST

The authors declare there are no competing interests.
